# Fixed-length haplotypes can improve genomic prediction accuracy in an admixed dairy cattle population

**DOI:** 10.1186/s12711-017-0329-y

**Published:** 2017-07-03

**Authors:** Melanie Hess, Tom Druet, Andrew Hess, Dorian Garrick

**Affiliations:** 1 Iowa State University, Ames, IA USA; 2 LIC, Hamilton, New Zealand; 3 University of Liege, Liège, Belgium; 4 Massey University, Palmerston North, New Zealand

## Abstract

**Background:**

Fitting covariates representing the number of haplotype alleles rather than single nucleotide polymorphism (SNP) alleles may increase genomic prediction accuracy if linkage disequilibrium between quantitative trait loci and SNPs is inadequate. The objectives of this study were to evaluate the accuracy, bias and computation time of Bayesian genomic prediction methods that fit fixed-length haplotypes or SNPs. Genotypes at 37,740 SNPs that were common to Illumina BovineSNP50 and high-density panels were phased for ~58,000 New Zealand dairy cattle. Females born before 1 June 2008 were used for training, and genomic predictions for milk fat yield (n = 24,823), liveweight (n = 13,283) and somatic cell score (n = 24,864) were validated within breed (predominantly Holstein–Friesian, predominantly Jersey, or admixed KiwiCross) in later-born females. Covariates for haplotype alleles of five lengths (125, 250, 500 kb, 1 or 2 Mb) were generated and rare haplotypes were removed at four thresholds (1, 2, 5 or 10%), resulting in 20 scenarios tested. Genomic predictions fitting covariates for either SNPs or haplotypes were calculated by using BayesA, BayesB or BayesN. This is the first study to quantify the accuracy of genomic prediction using haplotypes across the whole genome in an admixed population.

**Results:**

A correlation of 0.349 ± 0.016 between yield deviation and genomic breeding values was obtained for milk fat yield in Holstein–Friesians using BayesA fitting covariates. Genomic predictions were more accurate with short haplotypes than with SNPs but less accurate with longer haplotypes than with SNPs. Fitting only the most frequent haplotype alleles reduced computation time with little decrease in prediction accuracy for short haplotypes. Trends were similar for all traits and breeds and there was little difference between Bayesian methods.

**Conclusions:**

Fitting covariates for haplotype alleles rather than SNPs can increase prediction accuracy, although it decreased drastically for long (>500 kb) haplotypes. In this population, fitting 250 kb haplotypes with a 1% frequency threshold resulted in the highest genomic prediction accuracy and fitting 125 kb haplotypes with a 10% frequency threshold improved genomic prediction accuracy with comparable computation time to fitting SNPs. This increased accuracy is likely to increase genetic gain by changing the ranking of selection candidates.

**Electronic supplementary material:**

The online version of this article (doi:10.1186/s12711-017-0329-y) contains supplementary material, which is available to authorized users.

## Background

Availability of single nucleotide polymorphism (SNP) genotypes allows the estimation of breeding values at a young age with higher accuracy than breeding values based on parent average [1]. Genomic prediction is routinely performed by fitting covariates representing SNP allele dosage, which putatively relies on linkage disequilibrium (LD) between SNPs and quantitative trait loci (QTL) to estimate the QTL effects [2, 3]. Accuracy of genomic predictions improves when LD between SNPs and QTL increases, i.e. by increasing SNP density [4]. A haplotype block (haploblock) defines a region of the genome that comprises a set of neighboring genetic markers (i.e. SNPs), whereby their phased alleles are likely inherited together. A haplotype allele is a combination of phased SNP alleles that are present in a haploblock. Haplotype alleles are likely in higher LD with a linked QTL than the high minor allele frequency (MAF) non-coding SNP alleles that are typically used to construct SNP chips [5]. If the LD between haplotype alleles and QTL within the haploblock is higher than that between individual SNP alleles and QTL, the accuracy of genomic predictions that fit covariates for haplotype alleles is expected to be higher than the accuracy of genomic predictions that fit SNP alleles.

The prediction accuracy of haplotype models was shown to be influenced by the method used to divide the genome into haploblocks with both simulated [6, 7] and real [8] data. Simple methods to form haploblocks use measurements of length, such as centimorgans (cM) [9], base pairs (bp) [10] or number of SNPs [7, 8, 11], and apply these uniformly along the genome. These fixed-length haplotypes are easy to construct and their definition is not sensitive to the dataset that is used to construct them, unlike more complex methods [12, 13] that attempt to account for recombination hotspots and coldspots along the genome [14, 15].

Discarding SNPs with a low MAF is common practice when performing genomic prediction in order to reduce computation time and because of the low power to detect trait associations for SNPs with a low MAF [16, 17]. There are over 1 million (2^20^) possible haplotype alleles for a block of 20 biallelic SNPs, and although far fewer haplotype alleles are found in practice, many are typically observed at low frequency. Discarding these rare haplotype alleles will reduce computation time with little expected decrease in prediction accuracy, because the effect of rare alleles is shrunk towards zero in Bayesian linear regression models [18].

Cuyabano et al. [19] found that fitting covariates for haplotype alleles instead of SNPs increased the accuracy of genomic predictions when fitting a Bayesian mixture model but not when fitting a ridge regression best linear unbiased prediction (RR-BLUP) model. BayesA [20] fits all SNPs simultaneously and the effects of SNPs are assumed to be independent with a SNP-specific variance. Not all genomic regions are expected to be associated with a phenotype. BayesB [20] defines a parameter *π* and samples the effects of SNPs from mixture distributions, i.e. the effects for approximately 1 − *π* SNPs are sampled at each iteration of a Markov chain with the same assumptions as BayesA, and the remaining effects are assumed to be zero. BayesN [21] is a hierarchical extension to BayesB that assumes that some chromosome segments have non-zero effects and applies a local BayesB model only to the chromosome segments that are sampled to have an effect. Its hyperparameters include Π, i.e. the proportion of segments that are assumed to have no effect, from which it follows that a proportion of approximately 1 − Π segments are sampled to have non-zero effects at each iteration, and $\pi_{i}$, the segment-specific probability that a covariate within that segment has a zero effect. We hypothesized that BayesN would perform well when fitting covariates for haplotype alleles if each haploblock is considered as a window because it will estimate non-zero effects for those haplotype alleles that are in genomic regions (haploblocks) associated with the phenotype, and zero effects for covariates in all other regions.

Most studies using haplotypes to improve genomic prediction accuracy have focused on simulated datasets [7, 13, 22], or datasets consisting of a single breed [8, 12, 23]. The New Zealand dairy cattle population consists predominantly of Holstein–Friesians (HF), Jerseys (J), or their admixed descendants, known as KiwiCross (KX). Bulls used for artificial insemination (AI) include KX in addition to bulls that are predominantly (≥7/8) HF or predominantly (≥7/8) J; in New Zealand, only ~25% of semen straws, which are used to inseminate cows, are used on a cow of the same breed as the bull that provided the semen (i.e. HF, J or Ayrshire) [24], which results in most New Zealand dairy cattle being admixed in contrast to the situation in other countries [25]. This is the first study to quantify the accuracy of genomic prediction using haplotypes across the whole genome in an admixed population.

The objectives of this study were to evaluate the accuracy, bias and computation time of Bayesian genomic prediction methods that fit covariates for fixed-length haplotype alleles compared to SNP alleles. Fixed-length haplotype alleles (from 125 kb to 2 Mb) with varying allele frequency thresholds (from 1 to 10%) were fitted using BayesA [20], BayesB [20] and BayesN [21] models for genomic prediction when the training set included all breeds and validating the resulting predictions in later-born HF, J or KX cows not included in the training set.

## Methods

### Phenotype data

First lactation yield deviations (YD) [26] were provided by Livestock Improvement Corporation (LIC) for milk fat yield (Fat), liveweight (Lwt) and somatic cell score (SCS) for cows that were born between 1990 and 2011. Heritabilities of these traits in the New Zealand dairy cattle population are estimated at 0.28, 0.30 and 0.15, respectively [27]. Based on a six-generation pedigree, records for animals for which more than 1/16 of their genome originated from a breed other than Holstein, Friesian, J or Red Dairy Cattle (e.g. Ayrshire) were removed. All animals in small (<5 records) contemporary groups (same herd, parity, and calving season), outlier contemporary groups and outliers within a contemporary group were excluded. Outliers were defined as animals (or groups) for which records (or group mean) deviated more than 5 standard deviations (SD) from the mean for Fat and Lwt or more than 7 SD for SCS. Genotyped females with YD were used for training if they were born before 1 June 2008, and later-born genotyped females comprised the validation data. June 1 is the recognized start of the New Zealand Spring calving season. The number of animals in each training and validation set by breed is in Table 1.

**Table 1:** Numbers of records in training and validation sets used for genomic prediction

Breed^a^	Fat^b^		Lwt^b^		SCS^b^	
	Training	Validation	Training	Validation	Training	Validation
HF	9072	3354	3908	1464	9094	3358
J	5067	5854	2667	2331	5071	5860
KX	10,684	6125	6708	2436	10,699	6140
Total	24,823^c^	15,333	13,283^c^	6231	24,864^c^	15,358

^a^HF = predominantly (>7/8) Holstein–Friesian; J = predominantly (>7/8) Jersey; KX = admixed KiwiCross

^b^Yield deviation: Fat = Milk fat yield; Lwt = Liveweight; SCS = Somatic Cell Score

^c^Training was performed using pooled data across the three breed classes

### Genotypes and phasing

Genotype information was collected based on either v1 or v2 Illumina BovineSNP50 Beadchips [28] or the Illumina BovineHD Beadchip [29] for 58,369 dairy cattle born between 1960 and 2012 (46,614 females and 11,755 males). After filtering based on Hardy–Weinberg equilibrium (*P* < 1e−8), SNP call rate (<0.95) and excess Mendelian inconsistencies (>10), 37,802 mapped autosomal SNPs remained, which were phased using LINKPHASE3 [30]. SNPs that were associated with 35 putative map errors [30] were removed, leaving 37,740 SNPs. Some regions remained un-phased for some individuals, and these regions were phased with DAGPHASE [31] using the directed acyclic graph obtained from all haplotypes phased with BEAGLE [32].

### Haplotype construction

Haplotypes of five different lengths (125 kb, 250 kb, 500 kb, 1 Mb and 2 Mb) were constructed using the UMD 3.1 map of the *Bos taurus* genome (Genbank accession: DAAA00000000.2). Rare haplotype alleles were discarded based on their frequency in the training dataset at four different frequency thresholds: 1, 2.5, 5 or 10%. Discarding rare haplotype alleles results in the effect of these rare alleles being absorbed into the estimate of the mean. Five haplotype lengths assessed at each of the four frequency thresholds led to 20 scenarios being tested for each haplotype model.

### Genomic prediction models

Genomic prediction was performed using GenSel v4.73R [33], by fitting covariates for either SNPs or haplotype alleles in BayesA, BayesB or BayesN models. A single Markov chain Monte Carlo (MCMC) of length 41,000, including 1000 iterations for burn-in, was computed for each analysis to obtain posterior estimates of covariate effects, which were used to obtain direct genomic values (DGV) for validation animals, as described in the following section. Prior analysis showed that correlations and regression coefficients converged at this chain length.

#### BayesA

The SNP model and each of the 20 scenarios of the haplotype model (five haplotype lengths and four frequency thresholds) were fitted in BayesA for all traits, using the following model [20]:

$${\mathbf{y}} = {\mathbf{1}}\upmu + {\mathbf{Xh}} + \mathop \sum \limits_{{{\text{j}} = 1}}^{\text{k}} {\mathbf{z}}_{\text{j}} \upalpha_{\text{j}} + {\mathbf{e}},$$

where ${\mathbf{y}}$ is an N × 1 vector of YD, μ is the intercept, ${\mathbf{X}}$ is an incidence matrix of pairwise heterosis fractions between Holstein (H), Friesian (F), J and Red (R) breeds, defined as the product of the pedigree-based proportions of each of the two breeds for an individual, ${\mathbf{h}}$ is a vector of six heterosis effects, ${\text{k}}$ is the number of covariates for SNPs (SNP model) or haplotype alleles (haplotype model), ${\mathbf{z}}_{\text{j}}$ is an N × 1 vector of allele counts (0/1/2) at SNP ${\text{j}}$ (SNP model) or haplotype allele ${\text{j}}$ (haplotype model), α_j_ is the additive effect of that SNP or haplotype allele, and ${\mathbf{e}}$ is an N × 1 vector of identically and independently distributed residual effects with zero mean and variance $\sigma_{\text{e}}^{2}$, where the prior for $\sigma_{\text{e}}^{2}$ is a scaled inverse Chi square distribution with scale parameter $S_{\text{e}}^{2}$ and $\nu_{e}$ degrees of freedom. BayesA assumes that SNP or haplotype allele effects have identical and independent t-distributions with scale parameter $S_{\upalpha }^{2}$ and $\nu$ degrees of freedom.

#### BayesB

The SNP model and two of the 20 haplotype scenarios were fitted using BayesB. We selected two haplotype scenarios, i.e. the most accurate scenario based on BayesA across all breeds and traits, and a model that fitted a similar number of covariates as the SNP model. The BayesB model [20] can be written as:

$${\mathbf{y}} = {\mathbf{1}}\upmu + {\mathbf{Xh}} + \mathop \sum \limits_{{{\text{j}} = 1}}^{\text{k}} {\mathbf{z}}_{\text{j}} \upalpha_{\text{j}} \updelta_{\text{j}} + {\mathbf{e}},$$

where variables are defined as for BayesA, except that BayesB is a mixture model that assumes that some of the α_j_ have zero effect. This is defined by the binary variable δ_j_ that represents whether covariate ${\text{j}}$ was fitted in the model according to hyperparameter $\pi$, such that $\updelta_{\text{j}} = 1$ with probability $1 - \pi$, or $\updelta_{\text{j}} = 0$ with probability $\pi$. BayesA is identical to BayesB when $\pi = 0$. Various $\pi$ values, i.e. 0.2, 0.35, 0.5, 0.65, 0.8 and 0.95, were compared for all traits with the SNP and the two haplotype models to evaluate the sensitivity of BayesB to the assumed $\pi$.

#### BayesN

Only the SNP model and the two haplotype scenarios that were fitted for BayesB were fitted for BayesN for each trait. The model for BayesN [21] was:

$${\mathbf{y}} = {\mathbf{1}}\upmu + {\mathbf{Xh}} + \mathop \sum \limits_{{{\text{i}} = 1}}^{\text{w}} \mathop \sum \limits_{{{\text{j}} = 1}}^{{{\text{m}}_{\text{i}} }} {\mathbf{z}}_{\text{ij}} \upalpha_{\text{ij}} \updelta_{\text{ij}} \Delta_{\text{i}} + {\mathbf{e}},$$

where variables are defined as for BayesB, except that ${\text{w}}$ is the number of windows (represented by haploblocks for haplotype models) and ${\text{m}}_{\text{i}}$ is the number of covariates (SNPs or haplotype alleles) in window ${\text{i}}$. Parameter ${\mathbf{z}}_{\text{ij}}$ is an N × 1 vector of allele counts (0/1/2) at SNP ${\text{j}}$ in window ${\text{i}}$ (SNP model) or of haplotype allele ${\text{j}}$ in window ${\text{i}}$ (haplotype model), α_ij_ is the additive effect of that SNP or haplotype allele. SNP or haplotype allele effects were assumed to have identical and independent mixture distributions of zero with probability $\Pi$ and t-distribution with scale parameter $S_{\upalpha }^{2}$ and $\nu$ degrees of freedom with probability $1 - \Pi$. This approach differs from that of Zeng [21], who sampled covariates with a window-specific variance. Parameter $\Delta _{\text{i}}$ is a binary variable that represents whether covariates in window ${\text{i}}$ are sampled with the same assumptions as BayesB ($\Delta _{\text{i}} = 1$ with probability $1 - \Pi$) or with a zero effect ($\Delta _{\text{i}} = 0$ with probability $\Pi$). Several $\Pi$ values, i.e. 0.5, 0.8 or 0.95, were assumed to test the sensitivity of BayesN to $\Pi$. The GenSel implementation of BayesN fitted ${\text{k}}$ covariates per window, whereby ${\text{k}}$ is a user-defined parameter, therefore $\updelta_{\text{ij}} = 1$ with probability $1 - \pi_{\text{i}}$ and $\updelta_{\text{ij}} = 0$ with probability $\pi_{\text{i}}$ where:

$$\pi_{\text{i}} = \frac{{{\text{m}}_{\text{i}} - {\text{k}}}}{{{\text{m}}_{\text{i}} }},$$

and ${\text{m}}_{\text{i}}$ is the number of SNPs in window ${\text{i}}$.

Each BayesN SNP model was run twice, once with ${\text{k}}$ = 2, which is equivalent to fitting BayesB within a sampled window, and once with ${\text{k}}$ set to the maximum number of SNPs in a window (i.e. $\pi_{\text{i}} = 0$), which is equivalent to fitting BayesA within a sampled window. Haplotype models were run with $\pi_{\text{i}} = 0$, which is equivalent to fitting BayesA within a haploblock.

### Evaluation of prediction models

The training set for all genomic prediction models included all breed classes (HF, J and KX), but predictions of validation cows were evaluated separately for each breed class. The DGV were calculated for validation cows as:

$$\widehat{{{\mathbf{DGV}}}} = {\mathbf{Z}}{\hat{\mathbf{\upalpha }}},$$

where ${\mathbf{Z}}$ is the ${\text{N }} \times {\text{M}}$ matrix of allele or haplotype counts (0/1/2), ${\hat{\mathbf{\upalpha }}}$ is the ${\text{M }} \times 1$ vector of allele effect estimates and ${\text{M}}$ is the number of SNPs or haplotype alleles. Model performance was evaluated based on prediction accuracy, which was calculated as the correlation between YD and DGV, and prediction bias, which was the deviation from 1 of the regression coefficient of YD on DGV.

#### Bootstrap samples

Estimation of the accuracy and bias of genomic prediction from the entire validation set does not give an indication of the sampling error associated with the estimate; thus, standard errors were obtained from a single training analysis using 10,000 bootstrap samples of validation animals for each breed. Validation animals within a breed were sampled with replacement to obtain a sample that had an equal size to that of the validation set for that breed. The same bootstrap samples of validation animals were used for all scenarios and models. Prediction accuracy and bias were calculated for each bootstrap sample, and the estimate and standard error of these parameters for the validation set were the mean and standard deviation across bootstrap samples. Comparisons between models were obtained from paired t-tests of the 10,000 bootstrap samples, for which accuracies (or biases) were paired across each model for the same sample of animals. The *t* tests were one-sided when comparing the accuracy of a haplotype model to the accuracy of a SNP model because we were interested in testing whether haplotype models improved prediction accuracy over a SNP model, and two-sided otherwise. Significance was determined based on a *p* value threshold of 0.05.

#### Additional evaluation criteria

In addition to accuracy and bias of the models, the number of random effects fitted in the model (SNPs or haplotype alleles) and computation time were evaluated. The mean squared error of the model for the validation set of animals was also assessed.

### Potential impact of haplotype models on selection decisions

The Spearman rank correlation of DGV from all cows and the top 100 cows based on DGV were compared between the BayesA SNP model and the Hap250-1 model (250 kb haplotypes, fitting haplotype alleles with a frequency higher than 1% in the training set). According to DairyNZ [34], the top ~0.9% of cows are selected to be dams for the next generation of bulls in New Zealand. Therefore, the number of cows that were in the top 0.9% for both models was also reported in order to evaluate whether moving from a SNP model to a haplotype model is likely to impact selection decisions.

## Results

The number of SNPs in each haploblock varied across the genome (Table 2). The minimum number of SNPs in a haploblock was 1 for all haplotype lengths. The average number of SNPs per haploblock ranged between 2 and 30 and the maximum number ranged from 6 to 54.

**Table 2:** Mean and maximum number of SNPs per haploblock length

Haploblock length	Number of haploblocks	Number of SNPs per haploblock^a^	
		Mean	Maximum
125 kb	17,452	2	6
250 kb	9676	4	10
500 kb	4978	8	17
1 Mb	2514	15	31
2 Mb	1267	30	54

^a^The minimum number of SNPs in a haploblock was 1 for all haplotype lengths

### BayesA

#### Prediction accuracy and bias

**Fig. 1:**
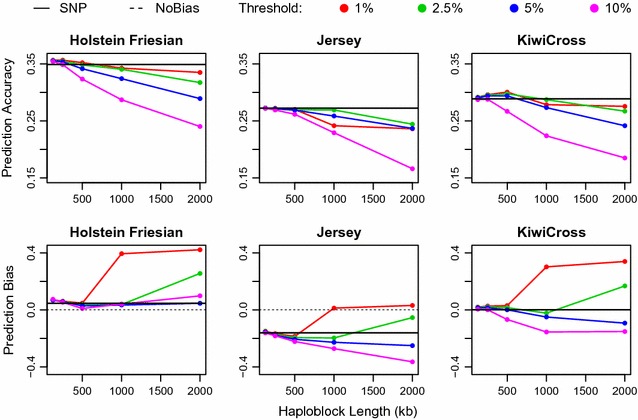
Genomic prediction accuracy and bias of milk fat yield with varying haplotype lengths and frequencies

Prediction accuracy and bias of each BayesA model are in Fig. 1 (Fat); Additional file 1: Figure S1 (Lwt), Additional file 2: Figure S2 (SCS), and Additional file 3: Table S1. Among the three traits, prediction accuracy was highest for Lwt, followed by Fat and SCS (see Additional file 3: Table S1), which is consistent with their heritabilities. Prediction accuracy was higher in HF than J for all three traits, whereas in KX it had an intermediate value for Fat and SCS but was highest for Lwt. Overall, the most accurate model used 250-kb haploblocks and a 1% haplotype allele frequency filter; more generally, models that fit short haploblocks (125 or 250 kb) tended to be more accurate and similarly or less biased than SNP models as shown in Fig. 1; Additional file 1: Figure S1 (Lwt) and Additional file 2: Figure S2 (SCS). Accuracy and bias were reasonably robust to change in frequency filter threshold at short lengths as shown in Fig. 1; Additional file 1: Figure S1 (Lwt) and Additional file 2: Figure S2 (SCS). Using haploblocks longer than 500 kb tended to decrease accuracy and increase bias of the haplotype model (i.e. they deviated more from 1), especially when using a higher haplotype allele frequency threshold as shown in Fig. 1; Additional file 1: Figure S1 (Lwt) and Additional file 2: Figure S2 (SCS).

#### Number of covariates and computation time

Table 3 shows the number of random covariates that were fitted in each BayesA model and the computation time in hours for each model, excluding the time to generate and filter the haplotype alleles. The number of covariates was similar across the three traits. The fastest models ran in 15 to 20 min and fitted only 650 to 700 haplotype alleles, depending on the trait (Table 3), but they were associated with a drastic decrease in accuracy and increase in bias as shown in Fig. 1; Additional file 1: Figure S1 (Lwt), Additional file 2: Figure S2 (SCS), and Additional file 3: Table S1. Computation times increased as the number of covariates increased (Table 3). The most accurate model for all three traits (250 kb haploblocks and a 1% haplotype allele frequency filter [Fig. 1; Additional file 1: Figure S1 (Lwt), Additional file 2: Figure S2 (SCS)] took approximately twice as long to run than the SNP model because it fitted approximately twice as many covariates (Table 3).

**Table 3:** Computation time and number of random covariates in haplotype and SNP BayesA models

Trait	Freq^a^	Number of random covariates					Computation time (h)^b^				
		125 kb	250 kb	500 kb	1 Mb	2 Mb	125 kb	250 kb	500 kb	1 Mb	2 Mb
Milk fat yield	SNP	37,226	37,226	37,226	37,226	37,226	13.1	13.1	13.1	13.1	13.1
	1%	56,590	64,724	70,380	56,534	32,520	22.8	23.5	24.7	20.0	11.3
	2.5%	51,889	53,482	47,378	29,343	13,460	21.3	19.7	16.8	10.4	4.8
	5%	46,283	41,737	28,324	12,291	3977	19.6	15.5	10.4	4.5	1.5
	10%	37,848	27,656	12,790	3255	646	15.2	10.8	5.0	1.4	0.3
Liveweight	SNP	37,356	37,356	37,356	37,356	37,356	6.6	6.6	6.6	6.6	6.6
	1%	56,595	64,634	70,218	56,164	32,117	11.0	13.1	13.3	9.9	5.7
	2.5%	51,839	53,204	46,797	28,756	13,050	10.2	9.6	9.2	5.2	2.4
	5%	46,163	41,467	28,040	12,198	4027	9.2	7.7	5.2	2.3	0.8
	10%	37,775	27,604	12,882	3354	707	7.8	5.4	2.6	0.7	0.2
Somatic cell score	SNP	37,229	37,229	37,229	37,229	37,229	13.0	13.0	13.0	13.0	13.0
	1%	56,630	64,730	70,375	56,521	32,516	21.4	24.4	27.2	19.7	11.1
	2.5%	51,934	53,488	47,385	29,348	13,464	23.1	20.8	16.7	10.9	4.7
	5%	46,326	41,746	28,329	12,296	3977	18.3	15.4	10.2	4.5	1.5
	10%	37,898	27,663	12,793	3254	645	15.1	10.7	5.0	1.3	0.3

^a^Frequency threshold for removing rare haplotype alleles. SNP refers to fitting covariates for SNPs rather than haplotype alleles

^b^Computation time for running the analysis on the training set containing all breeds with a chain length of 41,000

### BayesB and BayesN

#### Haplotype model choice

Two scenarios from the BayesA analyses were chosen to evaluate whether a BayesB or a BayesN model would further improve accuracy over the BayesA haplotype model, i.e. the scenario with 250-kb haploblocks that fitted only the alleles that had a frequency in the training dataset (Hap250-1) higher than 1% and the scenario with 125-kb haploblocks that fitted only the alleles that had a frequency higher than 10% in the training set (Hap125-10). Hap250-1 was selected because it had the lowest mean square error (MSE) for all three traits (see Additional file 4: Table S2); this scenario also had the highest accuracy and a consistently low bias (Fig. 1; Additional file 1: Figure S1 (Lwt), Additional file 2: Figure S2 (SCS)]. The Hap125-10 model was selected because the number of haplotype alleles was similar to that of SNPs (Table 3), and could be used to evaluate whether it would be better to fit SNP or haplotype alleles if the number of covariates had to be constrained. The MSE of the BayesA Hap125-10 model was less than or equal to that of the SNP model for all three traits (see Additional file 4: Table S2).

#### Prediction accuracy

The accuracy of the BayesN SNP model was similar when non-zero effects were sampled for all SNPs in a window or for two SNPs in a window (see Additional file 5: Table S3). Since we found that window size (125 kb, 250 kb, or 1 Mb) had very little impact on prediction accuracy for BayesN SNP models, only the results obtained by using 250-kb windows and sampling all SNPs per window were further evaluated.

A range of values for $\pi$ (BayesB) and Π (BayesN), collectively referred to as pi values, were evaluated to determine the values that led to the highest accuracies. Accuracies were essentially the same but decreased when pi values were so high that too few features were fitted, corresponding to pi values higher than 0.8 for most traits and breeds (i.e. fitting covariates for approximately 20% of the genome accounted for the effects of large QTL as well as the polygenic portion of the trait (see Additional file 6: Figure S3, Additional file 7: Figure S4), i.e. ~7000 covariates for the SNP and Hap125-10 models and ~12,000 covariates for the Hap250-1 model. In this paper, BayesB and BayesN results will be presented for a pi value of 0.5 because, in many cases, this value resulted in the highest or close to the highest accuracy.

**Fig. 2:**
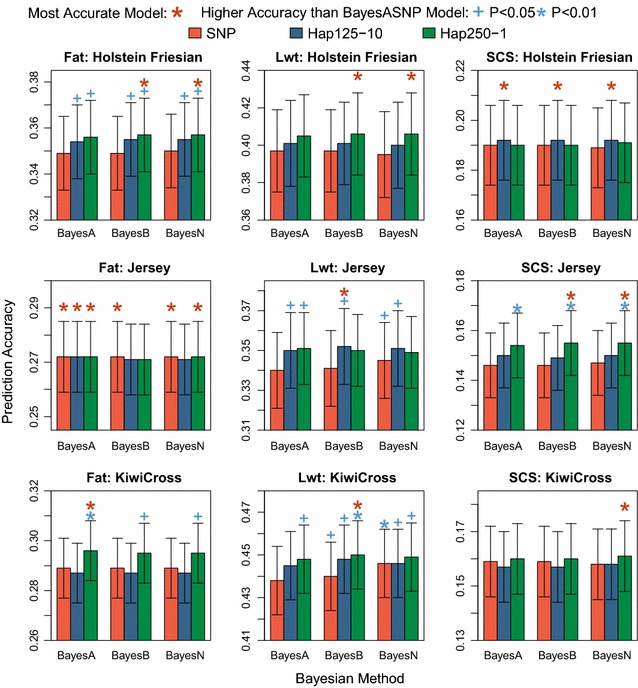
Genomic prediction accuracy of Bayesian SNP and haplotype models

The Bayesian method used (i.e. BayesA vs. BayesB vs. BayesN) had very little impact on prediction accuracy for both SNP and haplotype models (Fig. 2). Haplotype models were more accurate than the SNP model for all traits and breeds, except for Fat in J, which had a very similar prediction accuracy across all models. The 250-kb haploblocks tended to have higher accuracies than the 125-kb haploblocks but this difference was not significant (*P* > 0.30), except for SCS in J (*P* < 0.077) and Fat in KX (*P* < 0.001). Based on these results, the BayesA Hap250-1 model was chosen as a representative model for the comparison with the BayesA SNP model. Compared to the BayesA SNP model, the difference in accuracy of the BayesA Hap250-1 model was equal to 2.2 ± 1.1% (Fat; HF); −0.2 ± 1.2% (Fat; J); 2.6 ± 1.1% (Fat; KX); 2.1 ± 1.5% (Lwt; HF); 3.3 ± 1.7% (Lwt; J); 2.3 ± 1.1% (Lwt; KX); 0.1 ± 2.3% (SCS; HF); 5.5 ± 2.1% (SCS; J); and 0.2 ± 2.0% (SCS; KX).

#### Prediction bias

Prediction bias differed significantly from zero for all traits in J, for none of the traits in HF, and only for SCS in KX (Table 4). Compared to the BayesA SNP model, most models did not result in major changes in prediction bias, and those that were significant remained small. However, all significant changes in bias were beneficial, apart from the more negative bias when fitting the BayesN Hap125 model for SCS in J and KX.

**Table 4:** Prediction bias (standard error) of SNP and haplotype models for BayesA, BayesB and BayesN analyses

Trait^a^	Breed^b^	BayesA			BayesB (π = 0.5)			BayesN (Π = 0.5; π = 0)		
		SNP	Hap125^c^	Hap250^d^	SNP	Hap125^c^	Hap250^d^	SNP	Hap125^c^	Hap250^d^
Fat	HF	*0.05 (0.05)*	0.07 (0.05)	0.06 (0.05)	0.06 (0.05)	0.08 (0.05)	0.07 (0.05)	0.09 (0.05)	0.06 (0.05)	0.05 (0.05)
	J	−*0.16 (0.04)*	−0.16 (0.04)	−0.17 (0.04)	−0.15* (0.04)	−0.16 (0.04)	−0.16 (0.04)	−0.13* (0.04)	−0.18* (0.04)	−0.18 (0.04)
	KX	*0.00 (0.04)*	0.00 (0.04)	0.03 (0.04)	0.01 (0.04)	0.01 (0.04)	0.03 (0.04)	0.03 (0.04)	−0.01 (0.04)	0.01 (0.04)
Lwt	HF	−*0.04 (0.06)*	−0.03 (0.06)	−0.01 (0.06)	−0.03 (0.06)	−0.03 (0.06)	−0.01 (0.06)	0.00 (0.06)	−0.05 (0.06)	−0.03 (0.06)
	J	−*0.21 (0.05)*	−0.21 (0.04)	−0.18* (0.05)	−0.20* (0.05)	−0.20 (0.04)	−0.19 (0.05)	−0.15* (0.05)	−0.21 (0.04)	−0.21 (0.04)
	KX	*0.00 (0.04)*	−0.01 (0.04)	0.02 (0.04)	0.01 (0.04)	0.00 (0.04)	0.03 (0.04)	0.06 (0.04)	−0.02 (0.04)	0.01 (0.04)
SCS	HF	−*0.05 (0.08)*	−0.04 (0.08)	−0.04 (0.08)	−0.05 (0.08)	−0.04 (0.08)	−0.05 (0.08)	−0.04 (0.08)	−0.08 (0.08)	−0.08 (0.08)
	J	−*0.23 (0.07)*	−0.22 (0.07)	−0.18* (0.07)	−0.23 (0.07)	−0.22 (0.07)	−0.18* (0.07)	−0.22 (0.07)	−0.26* (0.07)	−0.21 (0.07)
	KX	−*0.18 (0.07)*	−0.20 (0.07)	−0.17 (0.07)	−0.18 (0.07)	−0.20 (0.07)	−0.17 (0.07)	−0.18 (0.07)	−0.23* (0.06)	−0.20 (0.06)

* Significantly different bias than the BayesA SNP model (*italics*) for that breed and trait (*P* < 0.05)

^a^Trait: Fat = Milk fat yield; Lwt = liveweight; SCS = somatic cell score

^b^Breed: HF = predominantly Holstein–Friesian; J = predominantly Jersey; KX = admixed KiwiCross (HF/J)

^c^Hap125 = haplotypes of length 125 kb, fitting only haplotype alleles >10% frequency in training data set

^d^Hap250 = haplotypes of length 250 kb, fitting only haplotype alleles >1% frequency in training data set

#### Number of covariates and computation time

In our study, computation time for all haplotype models was longer than for SNP models in all BayesB and BayesN analyses and was driven by the number of covariates that were fitted in each model (Table 5). BayesB models had a shorter computation time than the corresponding BayesA model, but BayesN models had a much longer computation time. Computation times for Fat and SCS were approximately double those for Lwt because the training set had approximately twice the number of records (Table 1).

**Table 5:** Number of random covariates (windows) and computation time for each model

Model^a^		Number of random effects^b^			Computation time (h)^d^		
		Fat^c^	Lwt^c^	SCS^c^	Fat^c^	Lwt^c^	SCS^c^
BayesA	SNP	37,226	37,356	37,229	13.1	6.6	13.0
	Hap125^e^	37,848	37,775	37,898	15.2	7.8	15.1
	Hap250^f^	64,724	64,634	64,730	23.5	13.1	24.4
BayesB	SNP	18,589	18,637	18,629	10.0	5.1	9.9
	Hap125^e^	18,899	18,831	18,954	13.6	6.2	13.9
	Hap250^f^	32,332	32,273	32,388	18.1	9.2	18.0
BayesN	SNP	17,748 (4701)	17,639 (4671)	18,254 (4805)	26.7	12.5	25.6
	Hap125^e^	18,451 (8264)	18,303 (8223)	18,711 (8344)	30.2	16.0	30.0
	Hap250^f^	31,596 (4737)	31,281 (4706)	32,103 (4809)	37.6	18.9	38.1

^a^SNP = SNP model with 250 kb windows

^b^Average number of SNPs or haplotype alleles fitted in each chain of the MCMC

^c^Fat = Milk fat yield; Lwt = liveweight; SCS = somatic cell score

^d^Computation time for running the analysis on the training set containing all breeds with a chain length of 41,000

^e^Hap125 = Haplotypes of length 125 kb, fitting only haplotype alleles >10% frequency in training data set

^f^Hap250 = Haplotypes of length 250 kb, fitting only haplotype alleles >1% frequency in training data set

### Potential impact of haplotype models on selection decisions

The Spearman rank correlation between DGV from the BayesA SNP model and BayesA Hap250-1 model was high (≥0.95) when considering all cows, but there was a considerable amount of re-ranking when considering only the top 100 cows for each breed and trait (Table 6). This re-ranking had an impact on which cows had DGV in the top 0.9%, which suggests that fitting haplotypes rather than SNPs will have an impact on which animals are selected as dams of sires.

**Table 6:** Rankings from the BayesA 250-kb haplotype model compared to the BayesA SNP model

Trait	Breed	r_S_ (All)^a^	r_S_ (Top 100)^b^	Top 0.9%^c^
Fat	HF	0.97	0.70	23/30
	J	0.97	0.68	41/53
	KX	0.96	0.55	36/55
Lwt	HF	0.96	0.57	10/13
	J	0.95	0.68	12/21
	KX	0.96	0.70	17/22
SCS	HF	0.96	0.58	21/30
	J	0.97	0.64	42/53
	KX	0.96	0.49	36/55

^a^Spearman rank correlation for all cows

^b^Spearman rank correlation for the joint set of cows that are in the top 100 cows for DGV from the SNP model or the top 100 cows for DGV from the haplotype model

^c^Number of animals with DGV in the top 0.9% for both the SNP model and haplotype model over the number of animals that are in the top 0.9% for the SNP model

## Discussion

Meuwissen and Goddard [35] predicted a promising increase in genomic prediction accuracy when increasing SNP density from ~30,000 SNPs to sequence-based SNPs. However these predicted results have not been observed in practice, i.e. only a slight increase in genomic prediction accuracy was found when fitting covariates for SNPs from the Illumina BovineHD panel (~777,000 SNPs) instead of the Bovine SNP50 panel (~54,000 SNPs) [36, 37], and little improvement or even a reduction in prediction accuracy was found when fitting sequence variants [38, 39]. Our study highlighted the potential of improving genomic prediction accuracy through the use of haplotypes. Fitting covariates for haplotype alleles rather than SNPs could increase prediction accuracy through improved ability to detect ancestral relationships between individuals (i.e. identity-by-descent), higher LD between causal mutations and haplotype alleles, or greater ability to capture short-range epistatic effects (i.e. between loci that are present within the same haploblock), and it is likely the result of a mixture of all three. The ability of a haplotype model to improve prediction accuracy depends on its prior assumptions, the method used to define haploblocks and haplotype alleles, SNP density, and the demographics of the training and validation sets.

### Haplotype parameters

#### Haploblock length

Villumsen et al. [7] evaluated the optimal haploblock length for simulated traits with heritabilities ranging from 0.02 to 0.30 and found that haploblocks of 1 cM gave the best results across all traits. For the genome of New Zealand dairy cattle, 1 Mb is equal to approximately 1.25 cM [40]. However, in our study, prediction accuracy was highest for much shorter haploblocks i.e. 250 kb (Fig. 1; Additional file 1: Figure S1 (Lwt), Additional file 2: Figure S2 (SCS)] and prediction accuracies of haplotype models were generally lower than those of the SNP model when haploblocks were longer than 1 Mb. This drop in accuracy is likely due to the large number of low-frequency haplotype alleles (i.e. due to low LD across large distances) that are generated from long haploblocks, which were removed in our analysis. If these rare haplotype alleles were not removed from the analysis, it is unlikely that prediction accuracy would be much affected because most of the rare covariates will not explain much of the genetic variance due to their low frequency and will therefore be shrunk to zero [18].

Prediction accuracies of haplotype models that used 500 kb or shorter haploblocks (less than eight SNPs per haploblock on average) were generally higher than those of the SNP model, particularly when haplotype alleles with frequencies lower than 1% were removed from the training set. Other studies have evaluated the performance of haploblocks defined by the number of SNPs (e.g. two or four SNPs per haploblock), mostly using simulated data. Simulation studies using a similar density to that used in our study (approximately 12.5 SNPs/Mb vs. an average of 15 SNPs/Mb), found that the optimal haploblock length ranged from 5 to 10 SNPs (i.e. 0.4 to 0.8 Mb) per haploblock [6, 7], which is slightly longer than the haploblock length that gave the highest prediction accuracy in our population. This difference in optimal haploblock length is likely due to the assumed simulation parameters, which deviate from the true values of these parameters in our dataset; based on simulated data, Villumsen et al. [7] demonstrated that the optimal number of SNPs in a haploblock depends on the distance between SNPs, the extent of LD and the population structure. Thus, the optimal haplotype length for an analysis needs to be evaluated for each dataset independently and by taking the purpose of the analysis (i.e. shorter for QTL mapping or longer for genomic prediction [11]) into account.

#### Haplotype allele frequency threshold

When using ~50 k SNPs to create haplotypes, the number of covariates to estimate is often much larger than the number of SNPs, which increases computation time, as shown in Table 3. In previous studies, the number of covariates that need to be estimated was reduced by removing SNPs before generating the haplotype alleles [11, 12] or by fitting covariates only for haplotype alleles in regions that have known or putative QTL, along with a residual polygenic effect [9, 12]. When appropriate filtering is performed, the resulting accuracy of genomic prediction can be equal to, or even higher than that reached by using all haplotype alleles, as shown by Cuyabano et al. [12].

When haplotype alleles are fitted as random effects, as in BayesA, BayesB and BayesN, the estimated effects are shrunk relative to the variance assumed for that allele (i.e. $\sigma_{e}^{2} /\sigma_{{\upalpha_{j} }}^{2}$) [18, 20]. A haplotype allele with a low frequency will be shrunk more than another allele with a similar effect but with a moderate frequency. As expected, due to the polygenic nature of the traits in this study, removal of rare haplotypes for the shorter haploblocks had little impact on prediction accuracy for frequency thresholds below 5% and haploblocks that were 500 kb long or less, which confirmed that filtering based on haplotype allele frequency is a suitable method to reduce computation time (Table 3) with little loss in accuracy when the haploblocks have an appropriate length for the dataset.

### Bayesian models

Genomic prediction accuracy depends on the genetic architecture of the trait and on whether prior assumptions of the model appropriately account for the number of loci that affect the trait and the distribution of their effects [41, 42]. We selected BayesA [20] to identify the impact of haploblock length on genomic prediction accuracy because it provides a higher prediction accuracy than the Bayesian equivalent of GBLUP, BayesC0 [43], when a trait is influenced by large effect QTL [20], such as for Fat and Lwt [44, 45]. Although SCS is known to be a very polygenic trait [46], suggesting that BayesC0 may be more appropriate, Habier et al. [47] found that BayesA resulted in a higher prediction accuracy than GBLUP for SCS in North American Holstein bulls. Thus, BayesA was expected to be a suitable model for all traits evaluated in this study.

Cuyabano et al. [19] obtained higher prediction accuracy when fitting haplotype alleles rather than SNP alleles in genomic prediction models such as the Bayesian mixture model BayesR [37], however this improvement was not observed when fitting a Bayesian GBLUP model. BayesR assumes that SNP (or haplotype allele) effects come from a mixture of four normal distributions, such that most SNPs (or haplotype alleles) have little or no effect (i.e. are sampled from a distribution with small variance), while a proportion of the SNPs (or haplotype alleles) have a large effect (i.e. are sampled from a distribution with large variance). These results suggest that it is not appropriate to assume that haplotype allele effects follow a single normal distribution, such as in BayesC0, which further supports our choice of BayesA, in which SNP or haplotype allele effects are assumed to have a marker-specific variance.

We also evaluated the BayesB and BayesN models to determine which model would be more suitable for haplotype analyses and whether either model outperformed BayesA. When a large proportion of the variation in a trait is explained by a few large QTL, BayesA, which estimates non-zero effects for all SNPs or haplotype alleles, has been shown to be less efficient than models such as BayesB, which estimate non-zero effects for a proportion of the SNPs or haplotype alleles [20]. In our study, two Bayesian mixture models were evaluated in addition to BayesA: BayesB, which samples each haplotype allele regardless of the genomic region, and BayesN, which samples haplotype alleles within a genomic region jointly, based on whether or not the region is sampled in that iteration. As implemented in our study, the BayesN haploblock model can be considered as analogous to a BayesB model where the haploblock, rather than the haplotype allele, is sampled as being associated with the trait or not.

#### Performance of different Bayesian models

We found that BayesA, BayesB and BayesN models were all appropriate for genomic prediction that fitted covariates for haplotype alleles (Fig. 2). Our results were consistent with those of Zeng [21] at this SNP density, i.e. fitting two SNPs per window in a BayesN SNP model resulted in slightly lower prediction accuracy than fitting all SNPs per window (see Additional file 5: Table S3). However, it was surprising that BayesN did not result in higher prediction accuracy than BayesB for haplotype models; conceptually, covariates with non-zero effects estimated in an iteration are more likely to be associated with the trait in BayesN because all haplotype alleles within a haploblock are included or excluded simultaneously. In contrast, associations from BayesB analyses are more likely to be spurious because each haplotype allele independently has a zero or non-zero estimate sampled independently of the other haplotype alleles within the haploblock.

#### Computation time

In our study, computation times were much longer for BayesN than for BayesA and BayesB (Table 5), whereas Zeng [21] reported similar runtimes for BayesN and BayesB. When our dataset was tested with the C++ BayesN code used by Zeng [21], we obtained runtimes that were similar to those with BayesA but longer than those with BayesB. Thus, it may be possible to further improve computation time of BayesN when fitting covariates for haplotype alleles as implemented in our study by fixing $\updelta_{\text{ij}} = 1$ and only sampling $\Delta$, rather than sampling δ_ij_ for each haplotype allele (with probability $1 - \pi = 1$).

Models that fitted haplotype alleles typically fitted a larger number of covariates than models that fitted SNPs, and therefore had longer runtimes. The development of a haplotype model for use in genomic prediction is appealing given the improvement in prediction accuracy when fitting haplotype alleles rather than SNPs. The BayesB Hap250-1 model had similar runtimes as the BayesA SNP model (Table 5) and equivalent or higher prediction accuracy for all traits (Fig. 2).

### Potential impact of haplotype models on selection decisions

Theoretically, improvements in accuracy will result in improved genetic gain in a population [48]; however, if this increased accuracy does not change the ranking of individuals, it is unlikely to have a substantial impact on realized genetic gain. In practice, only a small percentage of cows are selected to be dams of the next generation of sires [34]. Thus, re-ranking among the top cows may have an impact on which individuals are selected as parents of the next generation. The rank correlation of the top 100 cows from either the SNP or Hap250-1 models was evaluated and was much lower than that evaluated across all animals (Table 6). This was consistent with the substantial differences in the way cows would be selected as the top 0.9%. Considering the re-ranking of the top animals and the improvement in accuracy for haplotype models over SNP models that were observed in our study, genomic prediction that fits haplotype alleles is expected to result in higher genetic gain than genomic prediction that fits SNPs.

### SNP density

Increasing SNP density will influence the ability to differentiate sequence-resolution haplotype alleles within a haploblock: at the sequence level, all true haplotype alleles in the dataset can theoretically be identified, while at lower densities a single identified haplotype allele may represent two or more true haplotype alleles. This impacts the ability of a model to accurately estimate the breeding value of an animal for that haploblock because the effect of the identified haplotype alleles will be a weighted average of the effects of the underlying true haplotype alleles, in addition to prediction error. Incorporating genotypes at causal mutations into haplotypes will allow a more accurate estimation of haplotype effects compared to not having the causal mutations in the haplotype, and will improve the ability to detect short-range epistatic effects between loci that are contained within the same haploblock [49]. Therefore, increasing SNP density has the potential to improve genomic prediction accuracy when using haplotype models. However, increasing SNP density will increase the number of identified haplotype alleles [50], which will increase the number of rare haplotype alleles at a locus, and thus shrink the effect of these alleles towards zero [18]. This can potentially limit any improvement in prediction accuracy that would otherwise occur when increasing SNP density.

### Impact of training set

#### Training set size

Prediction accuracy declines when the size of the training data set decreases. Haplotype models are likely to be more sensitive to decreases in training data sizes because haplotype alleles that are present in a validation animal are less likely to be observed in a small training dataset than in a large training dataset. Haplotype allele effects can only be estimated for alleles that are observed in the training dataset, thus validation animals with many missing haplotype alleles are unlikely to be predicted with high accuracy. It is expected that at least 1000 phenotypic records are needed to accurately estimate haplotype allele effects [8].

The number of animals in the training set may also impact the optimal haploblock length, i.e. a small training dataset may result in shorter optimal haploblock lengths than a large training dataset. The ability of a haplotype model to provide accurate DGV depends on both the power to accurately estimate the effect of the haplotype alleles fitted in the model and the ability of those haplotype alleles to capture QTL effects and relationships between animals. Longer haploblocks generate a larger number of haplotype alleles than shorter haploblocks, and many of these are present at low frequency in the population (Table 3) and therefore there is little power to detect associations when the training dataset is small. Longer haplotypes also primarily capture more recent relationships, although if they become too long the relationship between parent and offspring or between full-sibs can be less than 0.5 [51].

#### Multi-breed training set

Our study used a training population that consisted of multiple breeds, as is the case in New Zealand genomic evaluations [52]. Training on each breed separately may lead to higher prediction accuracy in some cases, for example if the phase between a tagging SNP and a large QTL differs in each breed, or if some QTL only segregate in one breed [53]. Fitting covariates for haplotypes rather than SNPs may improve genomic prediction accuracy by capturing breed-specific effects if haplotype alleles around these QTL are specific to a breed. Kachman et al. [54] found that a training dataset that contained multiple beef breeds did not improve accuracy of genomic prediction using SNPs over a training dataset that contained the subset of animals that were of the same breed as the validation dataset. However, a combined training set of Danish, Swedish and Finnish Red cattle was found to increase genomic prediction accuracy using both SNPs [55] and haplotypes [19] compared to within-breed training and validation datasets. These studies [19, 53–55] suggest that the relationship between breeds, particularly around QTL, is an important factor in the success of genomic prediction using a multi-breed training set. De Roos et al. [56] evaluated the genomic relationship between New Zealand HF, New Zealand J and populations of Holsteins from the Netherlands and Australia. They found that phase was highly correlated among HF and J in New Zealand i.e. the correlation was higher than between New Zealand HF and their other Holstein populations, which indicates that it is appropriate to use a multi-breed training dataset for genomic prediction of New Zealand dairy cattle.

### Phasing accuracy

Performance of haplotype models depends on the ability to accurately phase the genotypes of training and validation animals because phasing errors will result in the generation of incorrect haplotype alleles. Animals that are closely related are expected to share more haplotype alleles than animals that are distantly related [51]. Thus, phasing accuracy is expected to be higher in datasets that contain closely related animals than in datasets with only distantly related animals [15]. Phasing methods, such as LINKPHASE3 [30], that take advantage of pedigree information can improve phasing accurately, particularly when there are close relationships between animals in the dataset, i.e. sire and multiple offspring. The dataset used for phasing in our study contained over 58,000 animals, including most of the sires that were used in New Zealand in the past 20 years, as well as pedigree information confirmed through genotyping. These animals were initially phased using pedigree information, then regions for which phase was not clear were phased using population haplotypes from BEAGLE, as described in [30]. Phasing accuracy is expected to be high in our dataset because it is a large dataset with closely-related animals and because we used a method that takes advantage of pedigree information.

### Fixed versus variable length haplotypes

Our study evaluated haplotypes that were based on a fixed length, in Mb, throughout the genome. It has been shown that recombination rates vary across the genome in many species [57], and that this variation is particularly large in dairy cattle [14], which suggests that the optimal haploblock length for genomic prediction may differ across the genome because recombination breaks down LD and can create new haplotype alleles. Another reason why optimal haploblock lengths may differ across the genome in domesticated plants and animals is that they have undergone artificial selection for production traits for many generations, which has resulted in some regions around production-related QTL undergoing selective sweeps. Methods to define haploblocks that take different recombination rates or LD patterns across the genome into account, termed “variable-length” haploblocks, may result in higher genomic prediction accuracy than fixed-length haploblocks. Various methods to define the limits of variable-length haploblocks from SNPs have been proposed, such as pairwise LD [12, 19], identity-by-descent (IBD) probabilities [11, 13], or fitting splines to a test statistic [58]. These methods are more complicated and time-consuming than fixed-length methods based on distance in Mb because, in addition, they involve the calculation of LD, IBD probabilities, or the fitting of additional models.

## Conclusions

Fitting covariates for fixed-length haplotype alleles rather than SNPs can increase the accuracy of genomic prediction up to 5.5%. Haplotype length and filtering based on haplotype allele frequency have a large impact on prediction accuracy and bias, and are therefore important parameters to optimize for the population and the analysis that is performed because non-optimized applications may decrease accuracy. In this dataset, shorter haploblocks (125 to 250 kb with on average two to four SNPs per haploblock) resulted in higher accuracies and generally lower biases than longer haploblocks (1 Mb or longer with on average at least 15 SNPs per haploblock), which had lower accuracies than the SNP model and were deemed too long for genomic prediction in the New Zealand dairy cattle population. A more stringent haplotype allele frequency filter tended to decrease prediction accuracy, particularly when haploblocks were long. The BayesA model that consistently gave the highest accuracy and lowest bias was the model that fitted 250-kb haploblocks with a 1% haplotype allele frequency filter.

The Bayesian model that was used for haplotype models (BayesA, BayesB or BayesN) had very little impact on prediction accuracy, as long as the pi values were less than 0.8 for the BayesB and BayesN models. Fitting short (125 kb) haplotypes with a high (10%) frequency filter resulted in equivalent or higher prediction accuracy than fitting SNPs with comparable computation time. The BayesA model that fitted 250-kb haplotypes with a 1% frequency filter performed well for all traits and improved accuracy up to 5.5% compared to the BayesA SNP model across breeds and traits. The BayesB model that fitted the 250-kb haplotype alleles with a frequency higher than 1% in the training dataset had a similar accuracy and bias as BayesA and BayesN models but a much shorter computation time. Comparing the ranking of the top animals from the SNP model to the haplotype model suggested that the improvement in accuracy obtained by using haplotype models would result in a difference regarding which individuals are selected as parents of the next generation. Further studies should assess the impact of constructing haplotypes that better capture the population structure, since such methods may result in improved genomic prediction models.

## Additional files

**Additional file 1: Figure S1.** Genomic prediction accuracy and bias of liveweight with varying haplotype lengths and frequencies.

**Additional file 2: Figure S2.** Genomic prediction accuracy and bias of somatic cell score with varying haplotype lengths and frequencies.

**Additional file 3: Table S1.** Prediction accuracy and bias for BayesA haplotype models.

**Additional file 4: Table S2.** Mean square errors for the BayesA models.

**Additional file 5: Table S3.** Accuracy of BayesN model with Π = 0.5 fitting two or all SNPs per window.

**Additional file 6: Figure S3.** Accuracy of BayesB models with varying *π* values.

**Additional file 7: Figure S4.** Accuracy of BayesN models with varying Π values.
