# Supplementary material for: Fixed-length haplotypes can improve genomic prediction accuracy in an admixed dairy cattle population
Source: Genet Sel Evol. 2017 Jul 3;49:54. doi: 10.1186/s12711-017-0329-y (PMC5494768; doi:10.1186/s12711-017-0329-y)
Supplement: Supplementary file 2 — Additional file 2: Figure S2. Genomic prediction accuracy and bias of somatic cell score with varying haplotype lengths and frequencies. [file 12711_2017_329_MOESM2_ESM.pdf]

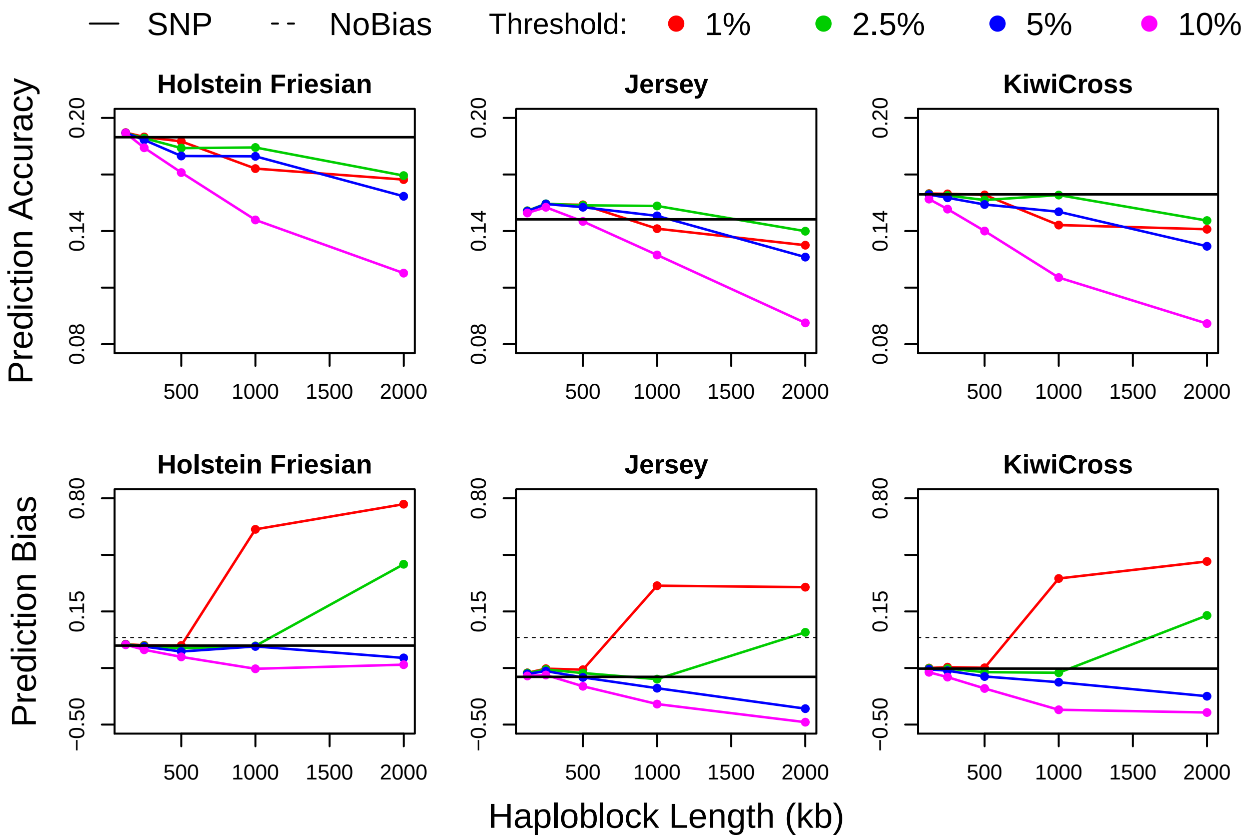

SNP
NoBias
Threshold:
1%
2.5%
5%
10%
Prediction Accuracy
Holstein Friesian
Jersey
KiwiCross
0.20
0.14
0.08
500
1000
1500
2000
Prediction Bias
0.80
0.15
−0.50
Haploblock Length (kb)
