# Supplementary figures and images for: Fixed-length haplotypes can improve genomic prediction accuracy in an admixed dairy cattle population
Source: Genet Sel Evol. 2017 Jul 3;49:54. doi: 10.1186/s12711-017-0329-y (PMC5494768; doi:10.1186/s12711-017-0329-y)

SNP

Hap125

Hap250

HF

J

KX

Max

Milk Fat Yield

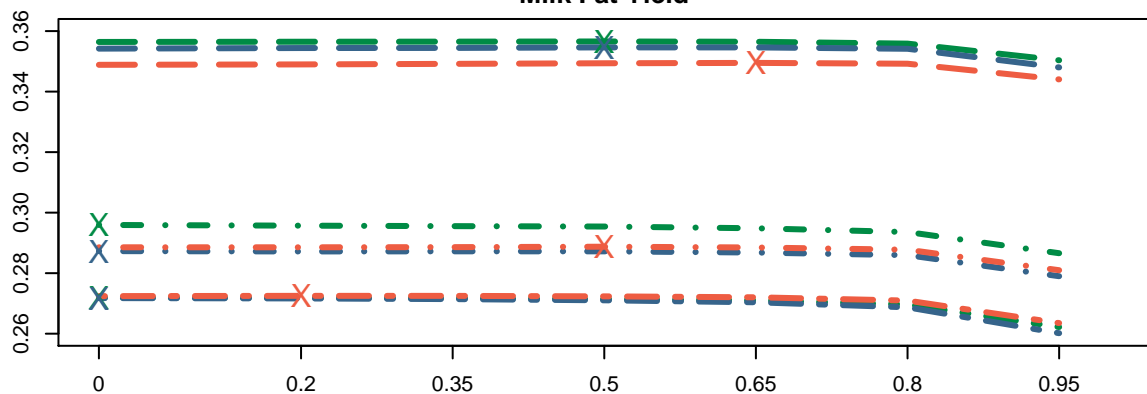

Liveweight

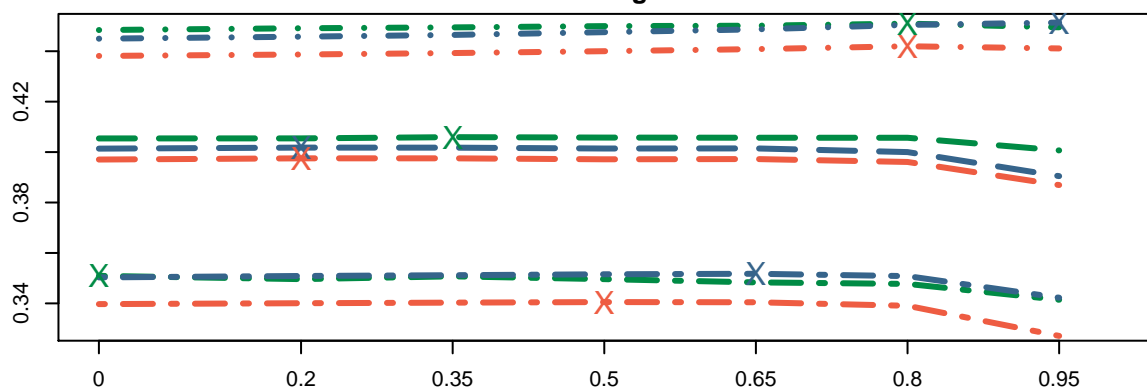

Somatic Cell Score

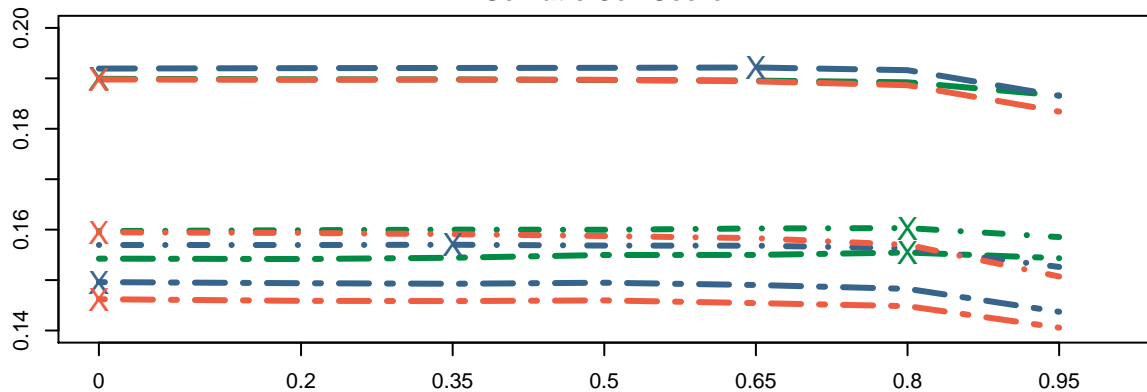

Pi

Prediction Accuracy

Supplement: Supplementary file 6 — Additional file 6: Figure S3. Accuracy of BayesB models with varying π values. [file 12711_2017_329_MOESM6_ESM.pdf]

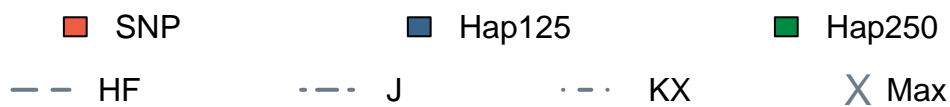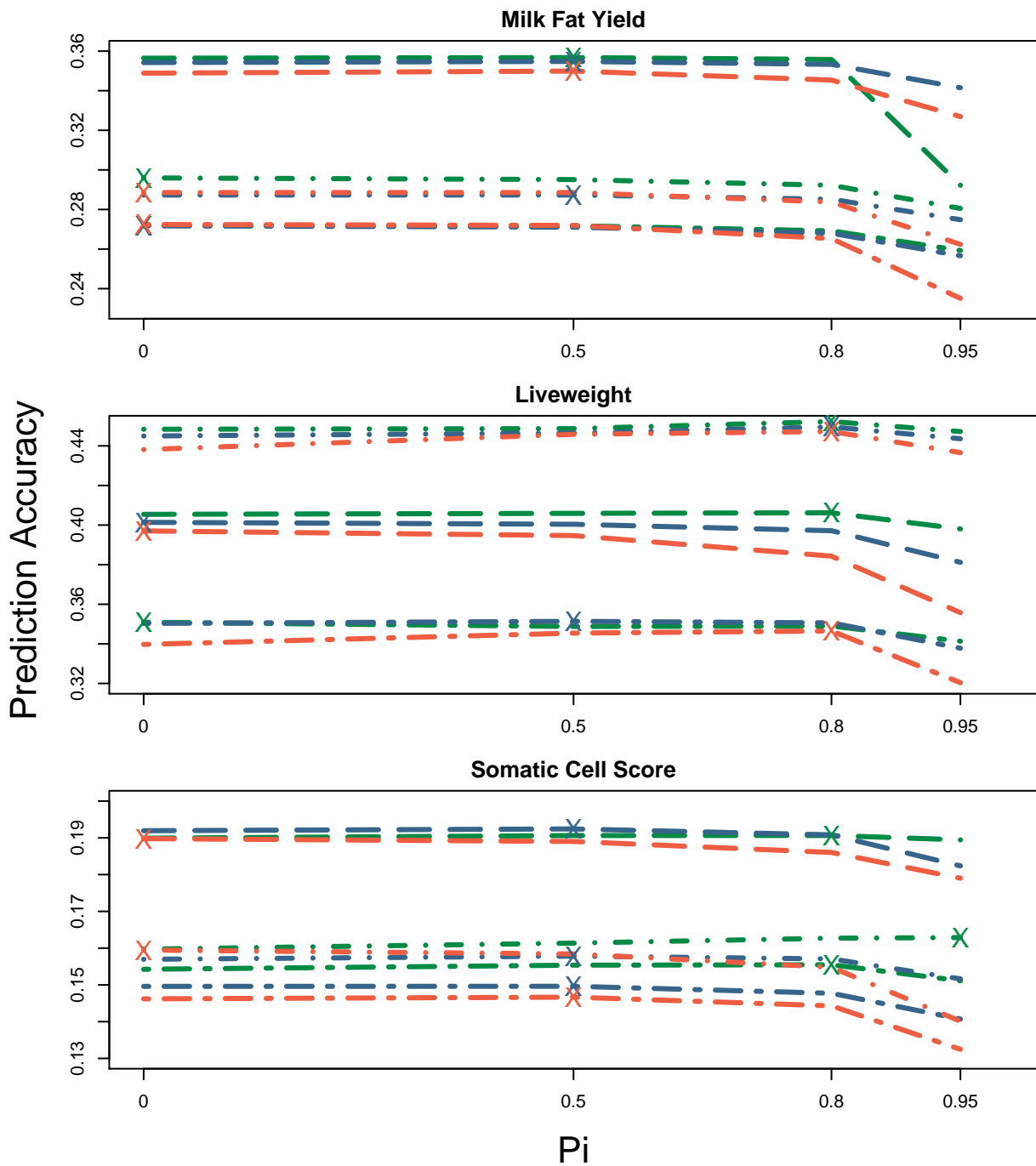

Supplement: Supplementary file 7 — Additional file 7: Figure S4. Accuracy of BayesN models with varying Π values. [file 12711_2017_329_MOESM7_ESM.pdf]
